# Medical Assistance in Dying (MAiD) for Canadian Prisoners: A Case Series of Barriers to Care in Completed MAiD Deaths

**DOI:** 10.1089/heq.2021.0117

**Published:** 2021-12-20

**Authors:** Peter Driftmier, Jessica Shaw

**Affiliations:** Faculty of Social Work, University of Calgary, Calgary, Canada.

**Keywords:** assisted dying/suicide, end of life, corrections, prison, medical assistance in dying

## Abstract

**Background:** As of August 2020, 11 patients who were federally incarcerated in a Canadian prison requested medical assistance in dying (MAiD), and three received it. This case study seeks to understand the process of care as described by physicians involved in each of the cases that resulted in MAiD.

**Methods:** During the summer of 2020, semistructured interviews were conducted with physicians involved in each known Correctional Service of Canada (CSC) MAiD case. Transcripts were summarized to illuminate details of the care process for each patient, highlighting barriers to patient-centered care.

**Results:** Each case took place in a different province. One MAiD provision took place in a prison hospital, and two provisions took place after the incarcerated patients were transferred to external community hospitals. Case summaries highlight the physicians' efforts and challenges in assuring patient-centered care.

**Discussion:** Physician experiences illuminate several barriers to care: CSC bureaucratic processes that forced longer wait times than typical for patients in the general public; challenges related to accessing release before MAiD application; knowledge of patient preference for location of death; concerns of voluntariness and confidentiality that are unique to CSC patients; and ethical considerations surrounding the presence of prison guards, police officers, and shackles at the time of assessment or provision. Reporting by the Office of the Correctional Investigator highlights additional challenges in these cases. Further inquiry is necessary to include the perspectives of prisoners and prison staff, and to consider how the evolution of new MAiD legislation will affect MAiD for prisoners.

## Introduction

Older adults are a growing proportion of Canada's federal prison population,^1^ requiring end–of-life care (EOLC) and medical assistance in dying (MAiD) to be growing realities for health care providers who interact with the Correctional Service of Canada (CSC).^‡^ CSC published Guideline 800-9 to provide guidance on how requests for MAiD ought to be addressed.^2^ It establishes additional processes that do not apply to patients in the general public, including screening for security information before initial assessment and the involvement of nonmedical personnel in parts of the decision making. The guideline proscribes settings external to CSC as the default for a MAiD death location.

Canada's federal prison ombudsman—the Office of the Correctional Investigator (OCI)—calls for stronger measures to limit access to MAiD unless the patient may first be granted release,^3^ and elsewhere asserts that MAiD should not be accessible to prisoners at all, citing the fact of incarceration as compromising voluntariness to consent to the procedure.^1^ Institutional stakeholders argue it is not a question of if, but how prisoners may access MAiD.^4^ Others argue that such safeguards found in CSC's current guideline may contribute to barriers that preclude patient-centered care, such as adding time to the process, potentially undermine voluntariness, and contravene international human rights norms of equivalent access to health care in prison as for the public.^5,6^

According to an August 2020 Access to Information and Privacy release from CSC, 11 official MAiD requests had been made by federally incarcerated patients to date, resulting in three completed MAiD procedures. According to this disclosure, all the three patients received MAiD in their requested setting. While each case has been briefly documented in reports from the OCI,^7,8^ the experiences of the physicians involved have neither been documented nor understood through the lens of delivering patient-centered care. This case study sought to understand how MAiD assessors and providers who have worked with CSC patients experienced the MAiD process in each of the known CSC MAiD cases to date.

## Methods

The initial research plan for this study, as approved by CSC and the research ethics board, was to interview CSC staff about MAiD. Interviews were scheduled for April 2020. In March, any and all research with CSC were suspended indefinitely due to the COVID-19 pandemic, including this study. The physicians who were interviewed for the cases, as presented in this study, were able to participate because they are not CSC staff.

This intrinsic case study describes the process of MAiD assessment and provision for three different Canadian prison MAID deaths as a unique phenomenon within broader access to MAiD services.^9^ Participants were recruited through a members-only forum of the Canadian Association of MAiD Assessors and Providers, of which the principal investigator has been a member since 2017.

Hour-long, semistructured interviews were conducted during summer 2020 over teleconferencing. Questions sought to elicit the physicians' experiences with EOLC and MAiD in the cases they were directly involved with, as well as their perceptions of patient-centered care for prisoners at end of life in general. The sample size (*n*=3) represents 100% of known MAiD deaths for federally incarcerated patients in Canada.

With no access to medical records or other internal documents, additional information about the cases was collected through Access to Information and Privacy Request and from annual reports of the OCI.

### Data analysis

Interviews were transcribed and then summarized by the same interviewer to focus on the details of the process of care.^§^ Each participant was emailed their case summary for comment or correction. In this article, each case has been labeled numerically by the date of provision, with each physician and patient numerically labeled by corresponding order (i.e., 1, 2, 3). Date of provision has been denoted by referencing the annual report in which the OCI documented the case.

To protect the privacy of participants and patients: names, geographic location of death, diagnostic and identifying health information, and exact dates of provision are withheld, although we note that each provision took place in a different province. Direct quotes are included to highlight the critical moment that each physician emphasized in their interview (Table 1).

**Table 1.** Quotes Highlighting Critical Moments

Case 1	*When we did the second assessment, the guards were in the room, and that's something that now that I have a few more gray hairs I would have done differently. I would have asked them to step aside at the beginning. The patient was handcuffed, and he asked during his assessments to be released from his handcuffs. Like I said, it was a difficult assessment, we were concentrated on other issues [including how being in prison affected eligibility and navigating provincial billing and approvals for a federal patient] so it didn't blow up in our faces. -Physician 1*
Case 2	*“Basically, the institution was his family” and the patient only requested the presence of his closest friend from the prison and his favorite nurse from the unit:*
	*They were both there, he was very close to them, he had a sunny bright room to die in, and everything in his room was organized the way he wanted it to. When he was ready, he said he was ready, and that's when we gave the medications. -Physician 2*
Case 3	*The MAiD provision was described as the most difficult case that the providing physician had ever been involved with:* *because there was no one that loved this man that was in that room. There was me, and the nurse, and a (police) officer—randomly—and two (CSC) guards. And everybody was uncomfortable. And it was just a different level of sad. Essentially, he wasn't dying alone, it was even worse. He was dying with his captors present. And I didn't understand why they needed to be in the room. He asked for music to be playing: some Johnny Cash. So, I'm playing Johnny Cash on my cell phone, trying not to cry, stroking his hand, because no one else was touching him, and pushing medication with the other. -Physician 3*

CSC, Correctional Service of Canada; MAiD, medical assistance in dying.

Interpretation of the cases was rooted in a commitment to patient-centered care: “care that is respectful of and responsive to individual patient preferences, needs, and values,” ensuring “that patient values guide all clinical decisions.”^10^ Thus, attention was paid to participant perceptions of when and how institutional or physician-centered decision making may have eroded or undermined patient autonomy, and instances of perceived respect for patient self-determination and dignity.

### Ethics approval

The University of Calgary Conjoint Faculties Research Ethics Board approved this study (REB17-0191).

## Results

### Case 1: 2017–2018 reporting period, physician 1 was an assessor

When Patient 1 first informed his parole officer that he wished to receive MAiD, he was advised to first apply for parole by exception (compassionate release). He was declined the application for such a release, at which point he submitted a request for MAiD.

Patient 1's first assessor was a physician within the prison. His second assessment took place at a community hospital by Physician 1, ∼1 month after his original request. The assessment took place while the patient was in handcuffs and with correctional officers in the room. At the request of the assessor, a consultant from the hospital's ethics committee supported them in an effort to ensure any potential pressures from the prison environment did not drive the request.

Physician 1 did not express awareness of patient preference for location of death. They had familiarity with Guideline 800-9, yet understood the policy as expressly prohibiting provision within prison. They described extra work that was involved to navigate provincial billing for a patient who was covered under a federal program. Patient 1 requested for the provision to take place 1 month later, to allow time for family to arrange to be present. Physician 1 was not present for the provision, but understands that the patient was not in handcuffs and that the guards remained outside the hospital room. Family, a chaplain, and the hospital ethics consultant were present. The CSC officers involved volunteered for this task, and they were debriefed by CSC afterward.

### Case 2: 2018–2019 reporting period, physician 2 was an assessor and provider

Patient 2 was receiving palliative care within a specialized CSC health care facility at the time of his MAiD request.

The providing physician commended CSC health care staff for their strong advocacy for Patient 2's expressed desire to die at the prison hospital. The process for allowing MAiD onsite required advocacy by the institution's nurses, taking unspecified “weeks, and weeks before they received permission from Ottawa to go ahead to contact us.” Officials in “Ottawa” may be a reference to Guideline 800-9's direction to Assistant Commissioner, Health Services (CSC's highest health executive) to approve requests for in-prison MAiD provision. Physician 2 described familiarity with the Guideline, but believed that CSC effectively banned any MAiD provision within custody following this case (the guideline has not been amended since its 2017 publishing).

The assessment took place in the institution simultaneously by two assessors, who returned together <2 weeks later for the provision. While the practice of simultaneous assessments and the presence of both assessors at provision are not standard across Canada, it is the standard practice of these two physicians.

Patient 2's reasons for choosing MAiD were described in terms of the advanced stage of illness: “he couldn't walk much anymore, he was in constant pain, on constant medications, his belly was huge… very much the same as anywhere else”—meaning having reached an advanced stage of illness. His desires to pursue MAiD provision in custody were related to him having lost all contact with his family of origin and community outside of prison. “Basically, the institution was his family” and the patient only requested the presence of his closest friend from the prison and his favorite nurse from the unit.

### Case 3: 2019–2020 reporting period, physician 3 was an assessor and provider

Patient 3 was assessed for MAiD while within the CSC institution, and both Physician 3 and another assessor were required by CSC to assess simultaneously with limited time available at this location. This simultaneous assessment was outside the normative practice standard, and beyond the comfort of this physician. Physician 3 believes that months had passed between the patient's request for MAiD and the assessment, and that the patient had been informed that MAiD could only take place through transfer to an external hospital. Physician 3 was not familiar with CSC Guideline 800-9.

Patient 3 was transferred to the local community hospital's cancer ward in the custody of two correctional officers. Officers were present at times when Patient 3 was assessed for voluntariness, in contrast to the providing physician's practice of asking others present to leave. Patient 3 declined the opportunity to invite or inform family of his death.

The MAiD provision was described as the most difficult case that the providing physician had ever been involved with. Following the provision, Physician 3 debriefed with all present, many of whom shared emotional “baggage” about death triggered by the event. This raised concerns for the physician that the presence of these nonhealth care staff was not only harmful and unnecessary for the patient but also for these additional staff involved.

## Discussion

### Health equity implications

Patient-centered care necessitates a displacement of institutional or physician-centered paternalism.^12^ MAiD assessors and providers assert that MAiD itself is by definition an expansion of patient-centered care, by expanding choice at end of life.^13^ The institutionalization of care that risks subordinating health care under carceral concerns compounds the necessity to attend to patient-centered care in EOLC.^14^

All the physicians spoke about the care and attention they made to assure quality of care to these three patients. They highlighted the constraints of long waiting times between steps that stretched far beyond those outlined standards of best practice and guidelines for nonincarcerated people. Physicians emphasized reduced access to communicate with the patients as a result of CSC procedures.

The cases highlight several barriers to patient-centered care, including challenges related to accessing release before MAiD, location of death, and role of external physician. All the cases took place following CSC's publication of its guideline on MAiD, which emphasizes that all avenues for release will be considered following a first assessment and according to standard CSC pre-release decision-making protocols.^2^

Patient preference of location for death was not always known by the attending physician. The physician in Case 2 knew of the patient's requested place of death to be in custody, while the others either did not know or did not say. The two physicians who said that they had familiarity with the CSC guideline misunderstood details about location of provision, believing that MAiD was not permitted to occur in custody. CSC's guideline states that MAiD will occur in a community setting, except “in exceptional circumstances, it may occur within custody at the request of the inmate” in a CSC “Treatment Centre or a Regional Hospital.”^2^

All three physicians believe that enforcing a transfer to a community hospital as the sole option of location for MAiD is a “forced transfer”**: a common barrier to patient-centered care in MAiD.^15^ Physician knowledge of location of death may be a barrier to provision of patient-centered care, should they discover discrepancies in reported preference. Finally, CSC's guideline details that the first MAiD assessment be conducted by an internal physician, which did not occur in Case 1 or Case 2.

The cases raise concerns related to the voluntariness and confidentiality of assessments that are unique to CSC patients. In Case 1, the individual had waited a year for the results of his parole by exception application, which was denied, before applying for MAiD. In Cases 1 and 3, the patients likely accessed the community hospital by temporary absence, meaning that if they withdrew consent for MAiD, they would return to prison.^5^ The CSC guideline outlines extra measures to evaluate voluntariness that are unique to the prison context before acquisition of a standard MAiD eligibility assessment,^2^ and the physician in Case 1 collaborated with the hospital ethics committee to assess for these prison-specific concerns.

There are ethical considerations surrounding the presence of prison guards, police officers, and shackles at the time of assessment and provision, which may erode dignity of the patient, may influence the ability of a patient to speak freely, impacting voluntariness, and can cause distress for patient and all involved.

Guards and shackles were present during the assessment of Case 1, despite the active participation of an ethics consultant. In Case 3, guards were present during assessment, with police also joining during provision. While CSC policy on the use of shackles and presence of nonmedical staff was not clear to participants, their presence during the assessment and provision of MAiD was perceived to be unnecessary, intrusive, and gross barriers to patient-centered care.

### Cases as described in other reports

The OCI^3,7,8^ has briefly documented these cases in annual reports, but the writing varies in detail related to process in each and their evidence is in the information reported directly from CSC to their office. Their review focuses on CSC, not the work of the medical professionals involved, stating the belief that the assessments and provisions of MAiD were conducted “compassionately,”^8^ and “professionally and with due consideration” to eligibility criteria for MAiD in Canadian law.^3^ However, in Cases 2 and 3, they report “a series of errors, omissions, inaccuracies, delays, and misapplications of law and policy,” without providing details about what these errors may be,^8^ and much of which we cannot confirm.

However, in our interviews, we found evidence of delays and violations of certain aspects of Guideline 800-9, such as Case 1's assessment for prison-specific pressures. Although “misapplications of law and policy” are not specified by the OCI, it is argued elsewhere that several aspects of Guideline 800-9 itself violate human rights norms for prison health,^6^ and denial of compassionate release may violate legislation surrounding parole.^17^

The OCI further describes the tendency for the Parole Board of Canada to deny compassionate release to applicants who are at end of life and who no longer pose undue risk to society as potentially contributing pressure to seek MAiD in these cases.^3,7,8^ Denial of parole had taken place in Cases 1 and 2. However, the physician in Case 1 offered relevant details that were missing from the OCI's report: that the patient had originally requested MAiD, but was suggested by staff to instead pursue release.^7^ In contrast, the physician in Case 2 described the patient's wish to receive MAiD in the CSC facility, a detail not included in the OCI report, which instead describes the patient's prior application and appeal to access standard parole as well as expressions of interest in parole by exception.^3^

The OCI described Patient 3 as having Dangerous Offender status whose “prospects for release, even considering the advanced stages of his illness, were minimal.”^3^ This patient did not apply for release before requesting MAiD.

### Knowledge gaps and future directions

Thorough discussion of the international landscape of assisted dying in prisons and ethical questions about voluntariness of MAiD and incarceration are beyond the scope of this article.^††^ Although there has been a pilot project examining the views and experiences of prisoners about MAiD and EOLC,^18^ these first-voice perspectives are paramount and require further exploration.

Future research may respond to new MAiD legislation where expanding eligibility criteria might include where a mental disorder is the sole underlying medical condition (MD-SUMC).^19^ This will raise new ethical and health equity questions about MAiD for prisoners, for example, if mental illness is related to irremediable, intolerable suffering that is caused by imprisonment. Finally, since the current CSC guideline on MAiD deviates from federal legislation in several areas, attention ought to be given to ensuring that all institutional procedures meet the legal and ethical requirements of access to MAiD in Canada.

### Limitations

While the interviews offer insight into the process of prisoner care from the perspective of MAiD assessors and providers, and while they currently represent 100% of known completed MAiD cases for federally incarcerated patients in Canada, the results are not generalizable to future MAiD cases. The study could not include information from medical records or other internal CSC or health documents, and thus, interviews were not corroborated and thicker understandings of the process of care are still unknown. This study did not delve into the reasons that the eight other prisoner applicants were denied access to MAiD, nor answer definitively the degree to which the care processes hinder or assure compliance with Canadian MAiD law.

## Conclusion

The cases presented above provide insight into the process of care that surrounds MAiD for prisoners from the perspectives of assessors and providers, which sometimes differ from how the cases are described in OCI reports. The needs of prisoners ought to be considered in upcoming discussions about the expansion of MAiD eligibility criteria to include MD-SUMC. Further inquiry is needed to understand how incarcerated EOLC patients experience both the denial of release and the denial of MAiD. Research with CSC staff and prisoners could also provide important perspectives on how EOLC, including MAiD, is being and could be offered in federal correctional institutions.

Since having an intimate knowledge of CSC policy is outside the scope of practice of health care professionals who are not CSC staff, it is imperative that the corrections system upholds the rights of prisoners throughout the application process for release, assessment, and provision of MAiD. The disparities between the process of care that the three physicians witnessed and how MAiD is accessed in the Canadian public demonstrate inequity that is imperative for policy makers to rectify.

## Data Sharing Statement

In accordance with ethics agreements and to protect the confidentiality of participants and the patients whom they worked with, raw data (recordings and interview transcripts) will not be publicly available.

## Abbreviations Used

CSC: Correctional Services of Canada
EOLC: end-of-life care
MAiD: medical assistance in dying
MD-SUMC: mental disorder is the sole underlying medical condition
OCI: Office of the Correctional Investigator
SSHRC: Social Sciences and Humanities Research Council of Canada

## Notes

^‡^
Medical assistance in dying—where a physician or nurse practitioner administers or facilitates the self-administration of a series of medications causing death—has been legal in Canada for people suffering from grievous and irremediable health conditions since 2016.

^§^
Process of care refers to “an evidence-based action or intervention performed during the delivery of patient care [and reflects] health care facilities' ability to execute and comply with recommended best patient care practices.” ^11^

^*^
*Forced transfers are defined as “when a health care facility requires a person who requests assisted dying to go off-site to receive—or even be assessed for—(MAID).” ^16^

^††^
For an overview of assisted death in prisons in jurisdictions around the world, see Downie, Iftene, and Steves.^5^ For discussion of the ethics surrounding MAiD voluntariness in Canada's prisons, see the OCI and CHRC^1^, and Driftmier and Shaw.^6^
